# Acute Otitis

**Published:** 1899-12

**Authors:** 


### Acute Otitis.

The *Gazzetta degli ospedali e delle cliniche* recommends:

℞ Ichthyol, gr. 15.
Glycerin,
Aq. dest., aa gr. 112½.

M. Sig. A few drops of this mixture to be dropped three times daily into the ear.—*N. Y. Med. Jour.*

